# Effect of Physical Exercise on Taste Perceptions: A Systematic Review

**DOI:** 10.3390/nu12092741

**Published:** 2020-09-09

**Authors:** Alexandre-Charles Gauthier, Roseane de Fátima Guimarães, Khoosheh Namiranian, Vicky Drapeau, Marie-Eve Mathieu

**Affiliations:** 1 École de Kinésiologie et des Sciences de l’Activité Physique de la Faculté de Médecine, Université de Montréal, 2100 Edouard Montpetit Blvd #8223, Montreal, QC H3T 1J4, Canada; alexandre-charles.gauthier@umontreal.ca (A.-C.G.); rdf.guimaraes@umontreal.ca (R.d.F.G.); 2 Department of Health, Tehran University of Medical Sciences, Tehran P94W+M3, Iran; khoosheh.namiranian@gmail.com; 3 Centre de Recherche de l’Institut Universitaire de Cardiologie et de Pneumologie de Québec, Département d’Éducation Physique et Institut sur la Nutrition et les Aliments Fonctionnels, Université de Laval, 2300, rue de la Terrasse #2214, Québec, QC G1V 0A6, Canada; vicky.drapeau@fse.ulaval.ca; 4 École de Kinésiologie et des Sciences de l’Activité Physique de la Faculté de Médecine, Université de Montréal, Centre de Recherche du CHU Sainte-Justine, 2100 Edouard Montpetit Blvd #8223, Montreal, QC H3T 1J4, Canada

**Keywords:** exercise, physical activity, nutrition, taste perceptions, gustative perceptions, chemosensory

## Abstract

The effect of physical exercise on nutrition has gained substantial interest in the last decade. Meaningful results have been produced concerning the effect of physical exercise on different appetite hormones and food choice/preference. While it is well known that taste and nutrition are related, the relation between taste and physical activity has not yet been fully explored. This systematic review aims to provide a detailed view of the literature on physical exercise and its effect on taste perceptions. Five tastes were included in this review: sweet, salty, bitter, sour, and umami. Sweet taste intensity, sensitivity, and preference were increased by acute physical exercise, but sweet preference was reduced by chronic physical activity. Perceived intensity and sensitivity decreased overall for salty taste, but an increased preference was noted during/following exercise. Sour taste intensity ratings were decreased following exercise and preference was enhanced. Umami taste intensity and sensitivity increased following exercise and preference was decreased. No significant results were obtained for bitter taste. While evidence regarding the effect of exercise on taste has arisen from this review, the pre-testing nutrition, testing conditions, type of test, and exercise modality must be standardized in order to produce meaningful and reproducible results in the future.

## 1. Introduction

Food consumption, appetite, and desire to eat are intrinsically connected and primarily depend on energy homeostasis and the hedonic aspect of food; these factors drive food consumption through both hormonal and reward pathways [1]. Ultra-processed foods have the characteristics of being palatable and rich in sugar, fat, and salt [2]. A study published by Hall and colleagues showed that, when presented with an ad libitum ultra-processed food buffet, participants tended to eat approximately 500 kcal/day more than those exposed to a low-processed food buffet [3]. Food palatability correlates with higher energy intake and has been heavily linked with obesity. In addition, a preference for fat is associated with a higher likelihood of developing obesity within the overall adult population, while a preference for fat and salt combined is positively associated with the risk of developing obesity within the adult male population [4]. Food consumption related to palatability has a perception-based anchorage in which taste plays a role; thus, reduced taste perceptions may also promote energy intake [5]. A study conducted by Jayasinghe et al. found that the frequency of daily sweet food intake had a negative correlation with sweet taste intensity perception, meaning that the intake increased as the perceived intensity decreased [6]. The same principle applies for energy intake and absolute carbohydrate intake in relation to sweet taste intensity [6].

It is well established that obesity fosters many comorbidities, such as metabolic syndrome [7] and diabetes mellitus [8]. Fortunately, physical activity has been negatively correlated with the appearance of these conditions by actively promoting a negative energy balance and thus helps with bodyweight control [9]. Physical activity has also been shown to reduce hunger feelings, prospective food consumption, and plasma acetylated ghrelin levels, which directly dictate the quantity of food consumed onwards [10]. To date, many studies evaluating the effect of weight loss on taste perceptions have focused on physical activity paired with behavioral changes and nutritional interventions. For example, Umabiki and Sauer found a positive association between bodyweight loss and sweet and sour taste perceptions, but the independent role of exercise in these changes has not yet been reported [11,12]. Although it has been determined that physical activity has an impact on appetite and food consumption, a growing body of research has studied the link(s) between physical activity and taste. A study published in 2019 unveiled many protective effects and treatment effects of physical activity on basic senses, including taste [13]. In fact, this study suggested that high physical activity levels are positively associated with higher taste sensitivity in older populations [13]. A better understanding of the impact of physical activity on taste perceptions will provide important evidence regarding the multifactorial effect of physical activity on overall energy metabolism, sensory perceptions, and nutrition.

This systematic review aims to highlight the state of the literature on the impact of physical activity and its structured form, i.e., exercise, regarding taste perceptions among humans. With the development of new technology with great potential regarding taste evaluation, the establishment of a meaningful relation between physical exercise and taste could create a new avenue for preventing and treating energy imbalances.

## 2. Materials and Methods

This review was conducted in accordance with the Preferred Reporting Items for Systematic Reviews and Meta-Analysis (PRISMA) statement guidelines [14].

Studies were selected if they included (1) protocols based on humans, children, or adults; (2) results that measured the effect of physical activity on gustation/taste perceptions; (3) study designs such as observational studies, experimental studies, and experimental mixed-model studies. Studies were not selected if they included (1) only food preference as a measurement for taste, given that food preference is too broad of a concept, or (2) solely languages other than French or English. Moreover, all of the studies had to be fully completed and published; abstract-only, presentation-only, and unpublished studies were excluded.

Taste intensity is defined by the authors as the literal intensity of a tastant. It is a by-product of anchored sensory perceptions by which the one perceiving becomes aware, through his gustative organs, of the the tastant and its strength. It is usually correlated with the concentration of a substance and is often measured with scales rating the strength/power of the tastant within the drink. Taste intensity can also be related to the primary characteristic of a certain food/solution, such as the sweetness, saltiness, bitterness, etc. Taste sensitivity was defined as the absolute concentration threshold necessary for detection of the tastant in a certain food/solution. Taste sensitivity can also relate to one’s ability to discern a tastant when compared with a non-tasting solution, a solution containing another tastant, or a solution with a different concentration of the same tastant. Taste preference is strongly associated with the hedonic aspect of a single food/solution and was assessed by the authors using various parameters such as pleasantness, tastiness, overall liking, ideal concentration, and palatability. This factor is a subjective aspect that is highly situational and time-dependent.

### 2.1. Literature Search

In this work, 6 databases were searched: PubMed (1946–present), Embase (1974–present), Cab Abstracts (1973–present), PsycNET, CINAHL Plus with Full Text (1937–present), and Web of Science (1945–present). The last search was conducted on February 4, 2020. The following keywords were used for physical activity: “aerobic training” OR exercise* OR “physical activity” OR “physical training” OR “resistance training” OR sport* OR “strength training” OR “weight training” OR weightlifting OR “weight lifting”. For gustation, the following keywords were used: bitter OR bitterness OR flavor OR flavour OR gustation OR gustatory OR saltiness OR salty OR savory OR savoriness OR savoury OR savouriness OR sour OR sourness OR sweet OR sweetness OR taste OR umami.

### 2.2. Study Selection

**Figure 1:**
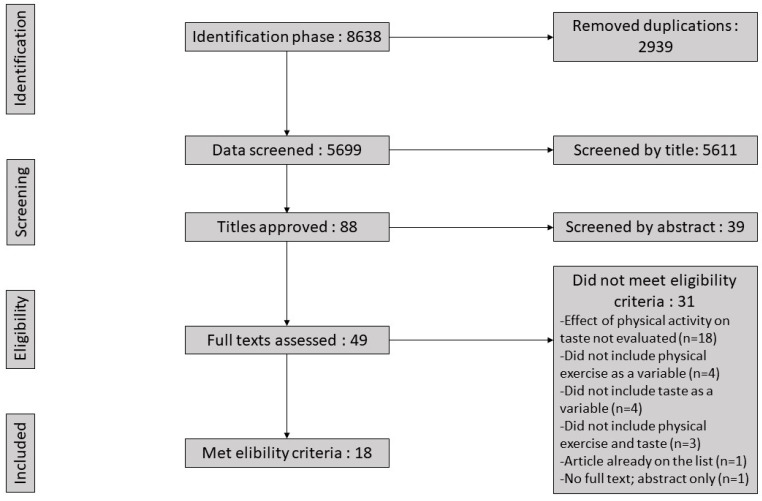
Systematic review flowchart.

The initial study selection was performed via title and abstract screening by two authors (A.-C.G., R.d.F.G.). Duplicates were removed. To be included in the final cut, studies had to implement some type of physical activity and taste perception test. A final selection was made by the reviewers (A.-C.G., R.d.F.G.) using full texts. Studies were selected in accordance with the eligibility and exclusion criteria. Any disagreements between authors were resolved internally by consensus. The number of articles included and excluded at each stage of selection are shown in the flow chart below (Figure 1. Systematic review flowchart).

### 2.3. Data Gathering and Analyses

The full texts of the articles that remained were carefully read and analyzed by A.-C.G. in order to extract the appropriate data from each text. K.N. independently verified and validated the extraction. In the case of discrepancy, a consensus was reached through discussion. The data, including detailed descriptions of each selected study, are summarized in Table 1, with the protocols given in Table 2.

### 2.4. Risk of Bias and Quality Assessment

The quality of the primary diagnostic accuracy of each article included in this study was assessed using the QUADAS-2 tool for assessing the risk of bias [15]. This tool consists of four key domains covering (1) patient selection, (2) index tests, (3) reference standards, and 4) flow of patients through the study and timing of the index test(s) and reference standard (“flow and timing”) [15]. Each domain was assessed in terms of the risk of bias, and the first three domains were also assessed in terms of concerns regarding applicability. The risk of bias was assessed by one reviewer (R.d.F.G.), and, in cases of uncertainty, a consensus was reached through discussion with two authors (A.-C.G. and M.-E.M.).

**Table 1:** Characteristics of included studies.

Author, Year	Country	Design	N	Age (Years)	Sample Characteristics	Intervention Details	Type of Taste Measure	Main Results
Chrystal et al., 1995 [16]	United States of America	Experimental (nonrandomized trial)	45	17–21 (experimental) 17–20 (control)	Division I varsity female swimmers (EX) and normal-weight females (CON)	Common conditions for all subjects: No caffeine, no MSG, no quinine, and no excessive sugar or excessive fat for 24 h before the test, with a low-fat, low-sucrose lunch 30 min before the test. No brushing teeth or chewing gum 30 min before the tests. Subjects were separated into 2 groups (exercise (EX) and control (CON)). Conditions for the CON group: 11 out of 17 of those who were initially chosen participated in all 4 tests; they completed different questionnaires on their eating habits, exercise habits, and other health-related traits in each session. Conditions for the EX group: Subjects were tested weekly for 4 weeks on the same day at the beginning of the athletic season. Subjects were then retested in the same manner at the end of the season for a total of 8 taste tests.	Visual analog scales.	The EX group and women who exercised more than 3 h/week (part of the CON group) all had decreased preferences for high sugar/fat stimuli when compared to CON (exercising less than 3 h/week). EX rated the sugar stimuli as significantly sweeter than CON but rated the fat stimuli as significantly less fatty. Significantly lower preference ratings were reported for the stimuli in the fall than controls in the winter (*p* < 0.01 and *p* < 0.05).
Kanarek et al., 1995 [17]	United States of America	Experimental (nonrandomized trial)	55	18–21	Healthy normal-weight female college students	Common conditions for all subjects: 34 out of 55 of those who were initially chosen were tested weekly for 4 weeks at the same time of day (11:30–12:00). Participants were asked to complete different questionnaires on their eating habits, exercise habits, sleep habits, and other health-related traits (TFEQ, POMS tests). Taste preference tests (tasting and rating samples of popcorn for palatability, saltiness, and fatness) were conducted in each session (4 total).	Taste preference with a hedonic rating scale.	Preference ratings for all samples of popcorn containing butter and salt were significantly higher for subjects who exercised more than 3 h/week compared to non-active and/or less-active groups (*p* < 0.01).
Nakagawa et al., 1996 [18]	Japan	Experimental	55	30.0 (mental task; bitter/sour) 25.3 (mental task; sweet) 28.0 (physical task; bitter/sour) 23.7 (physical task; sweet)	Males and females	Common conditions for all subjects: Subjects were separated into 2 groups (10:30 and 13:30) and were tested daily for one session (approximately 1 h). The study included 2 conditions: mental task (MT) and physical task (PT). Conditions for the MT group: POMS questionnaire and taste tests. The test was repeated after 40 min of conditions. Conditions for the PT group: POMS questionnaire and taste tests. The test was repeated after 10 min of cycling on a 100-W ergometer rotating at 60 rpm at a considerable speed.	Time intensity scale test (taste intensity scale).	Significant decreases in perceived intensity and total amount of taste were observed for sourness in relation to the PT condition (*p* < 0.001 and *p* < 0.01). The buffering capacity of saliva was enhanced following hard exercise.
Horio and Kawamura, 1998 [19]	Japan	Experimental (nonrandomized trial)	58	19–21	Healthy non-active university students	Common conditions for all subjects: No food or drinks 1 h before the tests. Taste tests (sucrose (sweet), NaCI (salty), citric acid (sour), MSG (umami taste)) just before exercise, 3 min after beginning exercise, and right after a 20-min exercise session. The study included 2 conditions: exercise (EX) and control (CON). Conditions for the EX group: Taste tests, 30 min of cycling on an ergometer bicycle at a pedaling rate of 50 rpm with the intensity adjusted as a function of the calculated MHR; HR, BP, and skin temperature were monitored every 3 min during exercise. Conditions for the CON group: Taste tests, 30 min of rest, followed by taste tests. The CON condition was implemented prior to the EX condition for half the group, and vice versa.	Taste preference with a hedonic rating scale and triangle test; absolute taste detection threshold test.	Preference for sucrose and citric acid increased significantly post-exercise compared to pre-exercise (*p* < 0.01 and *p* < 0.05).
King et al., 1999 [20]	United Kingdom	Experimental (nonrandomized trial)	16	21.3	Healthy male students	Common conditions for all subjects in three situations (pre- and post-exercise drink): Participants completed questionnaires on physical activity levels, eating habits, and mental and physical health and performed a VO_2_max test. Then, they were assigned to one of 3 conditions in each session, drank a sample of a solution, rated their sensory characteristics, and ran on a treadmill (approximately 50 min at 70% VO_2_max). Afterwards, they drank another sample of the same solution, rated their sensory characteristics, and drank a larger quantity of the same drink before an ad libitum test meal was presented to them, with various questionnaires about hunger levels, appreciation of the meal, etc.	Visual analog scales.	A significant effect of time was observed for perceived pleasantness of the sweet drinks (*p* < 0.01).
Leshem et al., 1999 [21]	Israel	Experimental (nonrandomized trial)	42	24.2 (control) 23.5 (exercisers)	Male students exercising (EX) 40–60 min 2–3 times a week and non-active male students (CON)	Common conditions for all subjects: No exercise the day before the experiment, a taste test in the morning (7:00–9:00) before eating or drinking anything other than water, a retest the following morning, and (12 h after exercise) an interview regarding their nutritional habits. Their preferences for salty or sweet snack items were considered based on added NaCl in soup and sugar in tea. Subjects were separated into 2 groups (exercise (EX) and control (CON)). Conditions for the EX group: Exercised in the evening (19:00–21:00) and had a second test within 30 min after the exercise session. Conditions for the CON group: Were asked to not consume anything 2 h before the evening taste test.	Preferred concentrations of salt and sugar were controlled by the participants; preferred snacks eaten were recorded.	The preference for salt increased significantly in each exercise group after exercise compared to the control (*p* < 0.05).
Wald and Leshem, 2003 [22]	Israel	Experimental (nonrandomized trial)	80	23.5 (males) 23.1 (females)	Students exercising both in basketball and scheduled aerobics (males and females)	Common conditions for all subjects: No coffee or alcohol 12 h before testing. Participants drank 100 mL of a novel drink and swallowed a capsule, either empty (placebo) or containing 200, 400, or 600 mg NaCl. The subjects were then divided into 4 groups equally participating in basketball or aerobic exercises (MHR @ 96.1% after 60 min). Taste preference tests were conducted after each exercise session. Four exercise sessions (EX) were conducted, but the first one was only for familiarization (FAM). The 3 other sessions were the same but included test trials.	Taste/flavor preference and intensity with line rating.	Flavor/taste preference was significantly influenced by sodium concentration in a bell-shape manner (*p* < 0.005) and was highly dependent on sweat levels (*p* < 0.005).
Horio, 2004 [23]	Japan	Experimental (nonrandomized trial)	44	19–21	Healthy non-active university students (males and females)	Common conditions for all subjects: No food 1 h before the tests, breakfast at 8 am. Taste tests (containing glucose, sucrose, D-sorbitol, stevioside, erythritol, and saccharin) were conducted just before exercise (or control) and right after 20 min of exercise. The study included 2 conditions: exercise (EX) or control (CON). Conditions for the EX group: Taste tests, 30 min @ 50 rpm with the intensity adjusted as a function of the calculated MHR on a bicycle ergometer, followed by repeated taste tests. Conditions for the CON group: Taste tests, 30 min of rest, followed by repeated taste tests. The CON condition was implemented prior to the EX condition for half of the group, and vice versa.	Taste preference with a hedonic rating scale.	Preferences for sucrose, glucose, stevioside, D-sorbitol, and erythritol all increased significantly (*p* < 0.01, *p* < 0.05, *p* < 0.01, *p* < 0.01, and *p* < 0.01) following the EX condition (pre- vs. post-EX).
Passe et al., 2004 [24]	United States of America	Experimental (nonrandomized trial)	50	36.4	Triathletes (males and females)	Common conditions for all subjects in 5 situations: Drinks reviewed included diluted orange juice (DOJ), homemade 6% carbohydrate-electrolyte sports beverage (HCE), commercially available 6% carbohydrate-electrolyte sports beverage (CCE), and water (W). The first session was an orientation session, with water used for testing. In the 4 subsequent sessions, participants were assigned to a new solution/drink with varying concentrations. Subjects were assigned to a drink first and then exercised 75 min @ 80–85% of the age-predicted MDOJHR. During exercise, at the 30- and 60-min marks, participants completed a hedonic rating scale and a sensory rating scale for the assigned drink.	Descriptive line scale and taste preference with a hedonic rating scale.	DOJ and W sweetness intensity ratings were significantly lower than those for HCE and CCE (*p* < 0.05) 60 min after exercise. DOJ, HCE, and CCE had significantly lower saltiness ratings than W (*p* < 0.05) 60 min after exercise. In terms of tartness intensity ratings, after 60 min of exercise, DOJ was considered the tartest and W was considered the least tart, with a significant difference in ratings between all drinks (*p* < 0.05).
Havermans et al., 2009 [25]	Netherlands	Experimental (randomized trial)	58	21.9	Undergraduate students (males and females)	Common conditions for all subjects: No eating or drinking 2 h before the experiment. All participants tasted and rated all 3 solutions upon arrival. Subjects completed a 15-min cognitive task followed by sensory ratings of gastrointestinal feelings. One of the 3 drinks (containing the solutions) was given to each subject before and after exercise. Subjects were separated into 2 groups (TASTE and DRINK) with 3 visits. TASTE: Participants tasted and spit out the drink 5 min before exercise, engaged in 30 min of treadmill exercise @ 80% of the calculated MHR, performed a sensory rating of gastrointestinal feeling, tasted and spit out the same drink, and performed 15 min of a cognitive task followed by taste tests of all three drinks with ratings. DRINK: Participants consumed all of the drink 5 min before exercise, engaged in 30 min of treadmill exercise @ 80% of the calculated MHR, performed a sensory rating of gastrointestinal feeling, consumed all of the drink, and performed 15 min of a cognitive task followed by taste tests of all 3 solutions with ratings.	Line scales (visual analog scales).	No significant results.
Narukawa et al., 2009 [26]	Japan	Experimental (nonrandomized trial)	35	24.7	Normal-weight male and female runners with healthy habits	Common conditions for all subjects: No food or drink 1 h before the taste test (except water). Participants were all running in a half marathon. Subject sensory evaluations were performed 1 h before the half marathon and immediately after (2 tests in total). The participants were asked to assess their physical fatigue before and after the half marathon. For sensory evaluation, the triangle test was used, which included one glass of sucrose solution and 2 glasses of distilled water provided in descending order of sucrose concentration.	Triangle test and taste detection absolute threshold test.	The absolute taste detection threshold of sucrose decreased following the half marathon, dropping from 11.9 ± 1.0 mM (*p* = 0.14) to 7.7 ± 0.8 mM (*p* < 0.001).
Passe et al., 2009 [27]	United States of America	Experimental (nonrandomized trial)	55	39.7 (males) 37.2 (females)	Normal-weight triathletes or runners (males and females)	Common conditions for all subjects: No physical activity and standardized nutrition the day before testing. Participants attend 6 visits, including one sedentary (SED) and 5 exercise (EX) testing conditions, with one sodium concentration for each session. SED: Blind-folded taste tests. EX: Blind-folded taste tests pre-exercise, aerobic circuit for 2 h @ 75% of the MHR, followed by taste tests at 60 and 120 min. Five test drinks, varying in sodium concentration (0,18, 30, 40, and 60 mmol/l), were evaluated in taste tests.	Visual analog scale and taste preference with a hedonic rating scale.	Exercise status and sodium level had a significant interaction with liking of the overall drink (*p* < 0.001). Exercise status had a significant main effect on the overall liking of the drink (*p* = 0.027). Exercise status and sodium level had a significant interaction with the liking of flavor and tartness (*p* = 0.046 and *p* = 0.034). The liking of sweetness, flavor, and tartness had a significant main effect for those in the exercise condition (*p* = 0.026, *p* = 0.035, and *p* = 0.004). Exercise status had a significant main effect on perceived intensity of tartness (*p* = 0.002).
Narukawa et al., 2010 [28]	Japan	Experimental (nonrandomized trial)	13	29.8	Normal-weight males and females in good physical health	Common conditions for all subjects: Arrival at 4 am in a fasted state (or at least no food 2 h prior). No drink or food 1 h before each taste test, but no restrictions during the session. Participants performed a 36-km mountain hike with 3 stops (16-, 25-, and 36-km marks) and 4 taste evaluations within 12 h. Participants tasted 2 solutions (100- and 300-mM sucrose solutions) 4 times (8) and rated their physical fatigue according to each distance.	Visual analog scale and a hedonic rating scale for palatability.	No significant results.
Wen and Song, 2010 [29]	China	Case control	900	40–75	Patients with confirmed cases of gastric cancer and controls without any cancer-related traits	Common conditions for all subjects: Subjects were selected from 2 groups (hospital patients and control group). All groups were asked to complete a questionnaire, and one-on-one interviews were conducted with each subject. The questionnaire included lifestyle habits, physical activity level, dietary habits, alcohol consumption, and other health-related sections. After interviews, a salt taste sensitivity threshold test (STST; 5 drops of NaCl solution were placed on the tongue) was performed for each participant.	Salt taste sensitivity threshold test (STST).	Significant correlation between physical activity and STST, with a Spearman rank correlation coefficient of 0.22 (*p* < 0.001).
Ali et al., 2011 [30]	New Zealand	Experimental (nonrandomized trial)	14	24.4	Healthy male recreational exercisers	Common conditions for all subjects: Controlled lifestyle and dietary factors 24 h before the test and a 3-h fast before each session. The group performed a sensory evaluation (including 3 sports drinks (high-carbohydrate/high-electrolytes, high-carbohydrate/low-electrolytes, low-carbohydrate/high-electrolytes) and water) 30 min before the exercise session. The other tests were conducted at 0, 30, 60, 90, and 120 min after the beginning of the exercise session. Participants performed a 60-min running session @ 70% of the MHR on a treadmill. All participants were tested weekly for 4 weeks (5 sessions in total, including the initial familiarization session).	Continuous analog scales.	Sweetness ratings were significantly higher during exercise compared to pre- and post-exercise (*p* < 0.001). Ratings for the high-carbohydrate/high-electrolyte drink decreased during exercise compared with post-exercise (*p* = 0.001). Ratings for the high-carbohydrate/low-electrolyte drink increased from pre-exercise to in-exercise (*p* < 0.001). Ratings for the low-carbohydrate/high-electrolyte drink increased from pre-exercise to in-exercise, and ratings were higher for pre- vs. post-exercise (*p* = 0.004 and *p* = 0.003). Sweetness ratings increased with exercise duration (*p* = 0.038). Saltiness ratings were lower in-exercise compared to pre-exercise (*p* = 0.003). Ratings decreased for pre- vs. in-exercise for the high-carbohydrate/high-electrolyte drink (*p* = 0.001). Saltiness ratings decreased with exercise duration (*p* < 0.01).
Martins et al., 2017 [31]	Norway	Experimental (randomized trial)	46	34.4	Sedentary healthy obese males and females	Common conditions for all subjects: 12-week exercise regimen (3 times/week) with monitoring of anthropometric measurements, body composition, maximal oxygen consumption, food preferences, and rewards before and after the end of the intervention (48 h after the last session). Fasting and postprandial subjective feelings of appetite and plasma concentrations of appetite hormones were measured before and after a standardized breakfast (every 30 min until 3 h) before and after the 12 weeks. Fat and sweet taste preferences and food rewards were measured using the Leeds Food Preference Questionnaire. Participants were separated into 3 groups: moderate-intensity continuous training (MICT), high-intensity interval training (HIIT), and 1/2 HIIT.	Leeds Food Preference Questionnaire.	Decreased explicit wanting, liking, and preference for savory relative to sweet foods (*p* < 0.001) was observed after 12 weeks.
Feeney et al., 2019 [32]	Ireland	Experimental (nonrandomized trial)	30	19–51	Healthy active (EX) and inactive (CON) males	Common conditions for all subjects: No strongly flavored food or drink 12 h before the test and no strenuous exercise 24 h before the test. Subjects were separated into 2 groups (inactive and active) according to their physical activity level. All participants were tested weekly for 2 weeks (2 sessions in total). Subjects were asked to taste 6 solutions with varying concentrations, to identify the substance, and to note the intensity and overall liking of the drinks (sucrose (sweet), citric acid (sour), sodium chloride (salt), quinine (bitter), and MSG (umami)). Participants were asked to complete different questionnaires (TFEQ and FFQ) on the last visit.	General labeled magnitude scale and general degree of liking scale.	The EX group was significantly better at identifying the umami taste compared to the CON group in general (*p* < 0.03). The EX group gave a higher rating for perceived intensity of high-concentration sucrose and high- and low-concentration umami than the CON group (*p* < 0.01, *p* < 0.01, and *p* < 0.05). The EX group also gave significantly lower ratings for the low-concentration carbohydrate taste and umami taste compared to the CON group (*p* < 0.01 and *p* < 0.01).
Josaphat et al., 2020 [33]	Canada	Experimental (nonrandomized trial)	12	18–35	Normal-weight non-athlete males	Common conditions for all subjects: No physical activity 24 h before the test and appetite sensation examination before sensory perception tests. In addition to the control group, subjects were separated into 2 groups with two visits: (1) EX9:40 and (2) EX10:30. EX9:40: 30 min of moderate to vigorous exercise on a treadmill at 70% of VO_2_max, followed by sensory perception tests and an ad libitum buffet-type meal. EX10:30: 90-min sedentary break before exercising, followed by sensory perception tests and an ad libitum buffet-type meal. Taste and smell tests were performed shortly after arrival and before lunch for both the CON and EX groups. For taste and smell tests, 10 liquid samples were considered.	Visual analog scales.	The timing of exercise in relation to a single ad libitum meal does not influence taste or smell perceptions or energy intake (*p* > 0.05 in all cases).

Abbreviations: MSG = Monosodium glutamate; TFEQ = Three-Factor Eating Questionnaire; POMS = Profile of Mood States; MHR = Maximum heart rate; HR = Heart rate; BP = Blood pressure; rpm = Revolution per minute; VO_2_max = Maximal oxygen uptake; MICT = Moderate-intensity continuous training; HIIT = High-intensity interval training; FFQ = Food frequency questionnaire.

**Table 2:** Protocol descriptions.

Author, Year	Variables Measured	Type of Exercise Description	Taste Experimental Protocol	Type of Taste Tests Description	Tests Duration
Chrystal et al., 1995 [16]	BMI, taste perceptions with hedonic and intensity scale, dietary restraint and disinhibition.	Exercise quantity was evaluated with a questionnaire and ranged from no exercise (control) to varsity college swimmers (exercise).	16 samples of milk were presented to the subjects with varying concentrations of sucrose (0%, 5%, 10% and 20%) and fat (0%, 3.5%, 10.5% and 37.6%) which were served every session (4 or 8).	The participants evaluated sweetness, pleasantness, and fatness on 160-mm on visual analog scales.	4 weeks, once/week or 8 weeks, once/week.
Kanarek et al., 1995 [17]	BMI, hedonic rating taste preferences, POMS results, menstrual cycle, dietary restraint and disinhibition and TFEQ.	Exercise quantity was evaluated with a questionnaire and ranged from no exercise to more than 3 h of exercise/week.	9 samples of popcorn were presented to the subjects with varying concentrations of both butter (3.3 g, 10.0 g, 30.0 g) and salt (0.0 g, 1.5 g, 4.0 g) which were served every session (4).	The preference test offered a hedonic scale rating from 1 (least pleasant) to 9 (most pleasant) for palatability, saltiness, and fattiness.	4 weeks, once/week.
Nakagawa et al., 1996 [18]	POMS results and time intensity test.	Exercise quantity was evaluated with POMS and self-examination. Participants also did a 10 min @100w for 60 rpm on an ergometer bicycle.	3 different samples of mixed water with sweet (sucrose; 2.63 ± 10^−1^ M), bitter (quinine sulfate; 1.82 ± 10^−5^ M), and sour (citric acid; 1.37 ± 10^−2^ M) taste test which were tested 2 times/session.	Time intensity scale test (taste intensity scale) filled on an online computer with a 30cm slide-type input device.	One session.
Horio and Kawamura, 1998 [19]	VO_2_max, hedonic rating taste preferences, absolute taste detection threshold, and heart rate.	Exercise modality was 30 min @50rpm with adjusted intensity on bicycle ergometer in function of the calculated MHR: exercising heart rate in beats per min. = (Maximum age-adjusted heart rate - resting heart rate) × 50% + resting heart rate.	5 different substances with different tastes (sucrose; sweet, NaCl; salty, citric acid; sour, caffeine; bitter, MSG; umami) with 5 varying concentrations, for a total of 25 drinks, were included in the taste preference test. The absolute taste detection threshold test had the same substances, but with 6 different concentrations. All tests were served before and after exercise and control condition for both tests and subjects had to detect the tastant-containing glass compared to a water-containing glass for every concentration.	The preference test offered a hedonic scale rating from 0 (extremely unpleasant) to 7 (extremely pleasant). The triangle test or the absolute taste detection threshold test consisted in the detection of the tastant-containing glass compared to a water-containing glass for every concentration.	2–3 days/visits.
King et al., 1999 [20]	BMI, VO_2_max, sensory and hedonic taste ratings, hunger profile, and energy expenditure.	Running on a treadmill for 45 min @70% VO_2_max.	3 conditions were present: exercise—bottled mineral water, exercise—low energy artificially sweetened fruit drink, exercise—high energy sucrose sweetened fruit drink. Each drink corresponds to a different session.	100-mm visual analog scales with which participants evaluated pleasantness, sweetness, palatability, and their desire to drink it.	4 sessions, 3 experimental.
Leshem et al., 1999 [21]	Body weight, hedonic rating taste preferences, and number of snacks eaten.	The intended sports program included fitness (10), basketball (6), and jogging (5) for 40–60 min in 3 groups. Exercise was evaluated in the morning before exercise, immediately after exercise, and on the following morning.	Participants would adjust the concentration of tomato soup by adding salt until they consider it “The most tasty”. Same applies for tea, except with sugar instead. Participants were also encouraged to eat freely from snacks that were both sugary or salty and the number of snacks eaten was recorded.	Adjusting concentration of salt or sugar until “The most tasty” concentration is attained (and then measured). Eating snacks freely and comparing which sugary or salty snacks were the most popular.	24-h period testing.
Wald and Leshem, 2003 [22]	Body weight, body weight loss, taste/flavor preference, and estimated MHR.	The intended sports program included basketball or aerobic exercises with an MHR @96.1% after 60 min.	Participants were divided into 4 groups equally and a standardized drink (root beer) which they were unfamiliar with was served every session with a capsule containing different NaCl concentrations (0, 200, 400, 600 mg).	Evaluation of flavor/taste preference and intensity with a line rating ranging from 0.0 to 10.0cm (from disgusting to excellent).	4 sessions.
Horio, 2004 [23]	Body weight, gustative perceptions and hedonic ratings, and fluid intake and predicted MHR.	Exercise modality was 75 min @80–85% of the age predicted MHR on bicycles ergometer, treadmills, and elliptical cross trainers.	4 different drinks with varying concentrations of carbohydrates, electrolytes, and other components were the solutions presented to the participants; diluted orange juice, homemade sport drink, commercial sport drink, and water. Each visit, participants were paired with one of the drinks (4).	The participants evaluated sweetness, saltiness, tartness, and thirst quenching on 100-point descriptive line scales. Liking of the overall drink, liking of flavor, liking of sweetness was measured with a 9-point hedonic scale.	5 sessions, 4 experimental.
Passe et al., 2004 [24]	Body weight, total body water, VO_2_max, hedonic rating taste preferences, and heart rate.	Exercise modality was 30 min @50 rpm with adjusted intensity in function of the calculated MHR: exercising heart rate in beats per min. = (Maximum age-adjusted heart rate - resting heart rate) × 50% + resting heart rate.	5 different sweet substances (sucrose, glucose, stevioside, sorbitol, erythritol, saccharin) with 6 varying concentrations, for a total of 30 drinks, were served before and after EX and CON condition.	Preference hedonic rating scale ranging from +3 (extremely pleasant) to -3 (extremely unpleasant).	2 days/visits.
Havermans et al., 2009 [25]	BMI, hedonic ratings for odor and taste, gastrointestinal sensory feelings, and MHR predicted by age.	Exercise quantity was evaluated with 30 min @80% of calculated MHR (207–0.7 * age; Gellish et al., 2007).	3 different drinks (mali-flavored or sala-flavored lemonade, cream soda) were smelled, tasted, and evaluated for hedonic ratings by each participant at the beginning and the end of the session (2). Afterward, subjects were given one of the 3 solutions for the whole session, which they consumed before and after exercise and the cognitive task (2).	100-mm and 200-mm line scales (visual analog scales) with hedonic ratings and sensory gastrointestinal feelings.	3 sessions.
Narukawa et al., 2009 [26]	Absolute taste detection threshold and subjective physical fatigue.	Participants ran a half marathon as fast as possible at their own pace.	6 different samples each containing 2 cups of distilled water and 1 containing sucrose were presented to the participants. Subjects had to detect the tastant-containing glass compared to the water-containing glasses for every concentration (40, 20, 10, 5, 2.5, and 1.25 mM).	The triangle test or the absolute taste threshold test consisted in the detection of the tastant-containing glass compared to water-containing glasses for every concentration.	One session.
Passe et al., 2009 [27]	Body mass, mean body mass loss, fluid intake, gustative perceptions and hedonic ratings, and MHR predicted by age.	Exercise modality was a 2-h circuit @70–75% of predicted MHR.	5 different drinks with the same flavor (lemon-lime), carbohydrates concentration (6%), and potassium (2.9 mmol/L), but different concentrations of sodium (0, 18, 30, 40, and 60 mmol/L), were tested for the sedentary condition. Every other exercise testing day, only one sodium concentrated drink was tested (for a total of 5 sessions).	The participants evaluated sweetness, saltiness, tartness, bitterness, flavor strength, and ideal saltiness on 100-mm visual analog scales. Liking of the overall drink, sweetness, tartness, flavor, and saltiness was measured with a 9-point hedonic scale.	6 visits, 2–3 sessions/weeks.
Narukawa et al., 2010 [28]	Body weight, taste sensitivity and palatability, subjective physical fatigue, number of steps, and energy consumption.	12-h hike with 3 pauses at 16, 25 and 36 km marks.	2 different drinks with varying concentrations of sucrose (100 and 300-Mm) were served to the participants. Participants evaluated each drink 4 times (including before the start of the hike) for a total of 8 measurements. Each test was followed by a 30-min break.	The participants evaluated intensity and the palatability on 100-mm visual analog scales. Subjects were also asked to rate the palatability on a hedonic scale ranging from -2 (unpleasant) to 2 (pleasant).	One session.
Wen and Song, 2010 [29]	BMI and STST.	Exercise quantity was evaluated with a questionnaire with 3 categories: never, 3 times or less/week, and 4 times or more/week.	10 different samples were presented to each participant ranging from 0.22 to 58.4 g/L of NaCl. Between every sample, mouth would be rinsed and there would be a 30-s break before the other test. Participants evaluated the solutions once.	Taste sensitivity threshold test (STST); participants needed to associate each concentration with certain food.	One session.
Ali et al., 2011 [30]	Body mass, sensory evaluations, RPE, and MHR predicted by age.	Exercise modality was 60 min of moderate to vigorous exercise session on the treadmill at 70% calculated MHR.	4 different drinks with key variances were served to the participants: (high-carbohydrate/high-electrolytes, high-carbohydrate/low-electrolytes, low-carbohydrate/high-electrolytes) or water. Participants were evaluating one substance each session (4).	The participants evaluated intensity of sweetness, saltiness, thirst-quenching ability, and overall liking on 100-mm continuous analog scales.	5 weeks, once/week.
Martins et al., 2017 [31]	BMI, VO_2_max, fat and sweet taste preference, PA level, dietary habits, and MHR.	12 weeks, 3 times/week with one of the following conditions: MICT (continuous exercise @75% of MHR for 250 kcal); HIIT (8s @85–90% of MHR and 12s of active recovery for 250 kcal); 1/2 HIIT (same protocol as HIIT, but for 125 kcal). Calories were measured according to VO_2_max. All sessions were performed on ergometers.	Participants were divided into 3 groups. Fat and sweet preferences were assessed with the Leeds Food Preference Questionnaire and a display of multiple images of different foods with varying energetic content and taste characteristics before and after a standardized breakfast and before and after the exercise intervention.	Leeds Food Preference Questionnaire protocol.	12 weeks, 3 sessions/week.
Feeney et al., 2019 [32]	BMI, body fat, general taste perceptions, dietary restraint, and disinhibition.	Exercise quantity was evaluated with a questionnaire and ranged from inactive (CON), defined as less than 1 structured exercise session/week, or active (EX), defined as at least 4 structured exercise sessions/week.	6 different substances of varying concentrations, ranging from low concentration to high concentration (total of 12 samples), of sucrose, acid citric, NaCl, MSG, quinine, and maltodextrin, were served every session (2). Participants were asked to identify the substance, to rate their intensity and their overall liking of the drinks. 30-sec break between substances and 2-min. break after 4 samples.	gLMS (general labeled magnitude scale), which is a validated scale for taste intensity, and gDOL (general degree of liking scale), which is a validated scale for liking of the stimuli.	2 weeks, once/week.
Josaphat et al., 2020 [33]	Body mass, body fat, waist circumference, VO_2_max, gustative and olfactory sensory perception, dietary restraint and disinhibition.	Moderate to vigorous exercise session on the treadmill at 70% of VO_2_max.	10 samples of milk with varying concentrations of fat (1%, 3.25%, 5%, 10%, and 15%) and sugary syrup (3%, 6%, 8%, 10%, and 12% carbohydrates) were served before and after the intervention. Ad libitum buffet-type meal composed of 14 liquid and solid items was served at lunch. The buffet was served in a private room with the same presentation and in controlled ambient conditions (odor, light, and temperature) on both visits. Appetite sensations were self-reported by the participants.	The participants evaluated sweetness, saltiness, fattiness, and liking on 100-mm visual analog scales.	2 days/visit.

Abbreviations: BMI = Body mass index; rpm = Revolution per minute; MSG = Monosodium glutamate; PA = Physical activity; TFEQ = Three-Factor Eating Questionnaire; POMS = Profile of Mood States MHR = Maximum heart rate; HR = Heart rate; VO_2_max = Maximal oxygen uptake; MICT = Moderate-intensity continuous training; HIIT = High-intensity interval training; STST = Salt Taste Sensitivity Threshold; RPE = Rate of perceived exertion.

## 3. Results

### 3.1. Study Selection

From the 5699 titles screened, 88 were approved based on their title, 49 were assessed using the full text, and 18 were analyzed and included in this systematic review. From the 18 studies, 17 had an experimental design, including non-defined [18], nonrandomized control trials [16,17,19,20,21,22,23,24,26,27,28,30,32,33], and randomized control trials [25,31]. One case control study [29] was also included. The number of participants ranged from 12 to 900, with a total of n = 1608 individuals. All articles included the effect of exercise on gustative perceptions and/or hedonic responses to different tastes: sweet, salty, bitter, umami, and/or sour. Intervention outcomes included different effects on taste intensity, taste preference, taste sensibility/detection threshold, or other hedonic responses.

### 3.2. Study and Intervention Characteristics

The studies originated from Canada [33], China [29], Ireland [32], Israel [21,22], Japan [18,19,23,26,28], the Netherlands [25], New Zealand [30], Norway [31], the United States of America [16,17,24,27], and the United Kingdom [20]. Most of the studies were conducted on healthy normal-weight males and females, but some studies included participants who were obese [31], clinically ill [29], or athletic [16,24,26,27].

Exercise intervention ranged from one session to a 12-week exercise program. Most of the exercise interventions were treadmill or ergometer sessions based on the maximal oxygen uptake [19,20,23,31,33] and/or calculated/predicted maximal heart rate [19,23,24,25,30]. Other interventions included organized sports [21,22], circuit stations [21,27], a half marathon [26], hiking [28], or questionnaires on physical activity levels [16,17,29,32]. In most cases, the subjects were trained in a controlled environment [18,19,20,23,24,25,27,30,31,33]; however, some participants trained outdoors [21,22,26,28] and/or were assessed on self-claimed activity specifications [16,17,29,32].

### 3.3. Taste Protocols and Tests

Most of the protocols used solutions with a predetermined quantity of ingredients, with salty [17,19,21,22,24,27,29,30,32,33], sweet [16,18,19,20,21,22,23,24,25,26,27,28,30,31,32,33], and bitter [18,19,27,32] as the most common tastes evaluated. Three studies used food with varying concentrations of salt, sugar, and/or fat for the taste evaluation [17,21,22], and one study used the Leeds Food Preference Questionnaire paired with images of food with varying energetic content and taste characteristics for taste evaluation [31].

In addition, 100- to 200-mm visual analog line/point scales were used for taste evaluation in nine [16,20,22,24,25,27,28,30,33] of the 18 studies, measuring variables such as intensity, overall liking, sweetness, saltiness, bitterness, tartness, sensory ratings, pleasantness, fattiness, and preference, among others. Hedonic scales ranging from 4 to 9 points were used in six studies [17,19,23,24,27,28], and one study used the Leeds Food Preference Questionnaire [31] for the taste evaluation test. Other tests included a time intensity scale test [18], a triangle taste test or absolute taste threshold test [19,26], a general labeled magnitude scale and a general degree of liking scale, which are both validated scales [32], and the salt taste sensitivity threshold (STST) test [29]. Only one study did not include any form of written/subjective taste evaluation with a scale or questionnaire [21].

### 3.4. Effectiveness of Intervention and Outcomes

Of the 18 studies considered in this systematic review, three offered no significant results regarding the impact of physical exercise on gustative/taste perceptions [25,28,33]. Perceived pleasantness, preferences, hedonic ratings, overall liking, liking of flavor of sweetness, sourness, bitterness, umami, or saltiness, which all relate to the hedonic aspect of the gustative response, were all significantly affected in 12 studies; a decreasing effect in four studies [16,30,31,32] and an increasing effect in eight studies [17,19,20,21,22,23,27,30] were found. Physical exercise increased taste intensity in five studies [16,24,27,30,32] and decreased taste intensity in two studies [18,30]. Taste sensitivity was increased in two studies [26,32] and decreased in one study [29].

While all five tastes were evaluated at least once in the 18 selected articles, saltiness and sweetness were the most commonly used tastes and produced the majority of significant results in terms of taste intensity, sensitivity, and preference [16,17,18,19,20,21,22,23,24,25,26,27,28,29,30,31,32,33]. Although some assessed the impact of physical exercise on savoriness/umami [19,32] and bitterness [18,19,27,32], tartness/sourness was the third most common taste evaluated with physical exercise in terms of perceived intensity, sensitivity, or preference [18,19,24,27,32].

### 3.5. Risk of Bias Assessment

The overall risk of bias was unclear or nonexistent. The risk of bias for all 18 studies is summarized in Table 3. Four studies were rated as having an unclear potential risk of bias for patient selection, while three studies were rated as having an unclear potential risk of bias for time and flowing. Regarding applicability concerns, which represent one of the methodological quality indicators (e.g., studies lacking information on inclusion criteria or randomization, allocation and outcome assessment concealment, and inadequate missing data handling), only five studies were rated as having an unclear potential risk of bias. For most studies, there was adequate information to make judgements about the methodological quality and risk of bias. Studies were not excluded due to these unclear risks of bias.

## 4. Discussion

### 4.1. Overall Results and Takeaways

The aim of this review was to determine whether physical activity (chronic) and exercise (acute) have a direct impact on taste perception. Following a systematic process, 18 studies were included. Of the 18 studies included in this systematic review, three did not supply significant results [25,28,33]. Taste intensity, preference, and sensitivity were all affected by exercise. Four studies showed decreasing trends towards the overall liking and preference of certain tastes [16,30,31,32], while eight studies showed augmentation trends following exercise [17,19,20,21,22,23,27,30]. Similar results were obtained for studies conducted on taste intensity and sensitivity: seven studies reported significant increases for both parameters and showed that physical activity increases taste intensity [16,24,27,30,32] and/or taste sensitivity [26,32] following an exercise session/program, with three studies showing significant decreases [18,29,30]. Table 4 offers a brief summary of these results in order to facilitate overall implications of physical exercise, in its acute and chronic forms, on taste perceptions.

### 4.2. Sweet

The effect of physical exercise on sweet taste received the most attention by far, with 16 out of the 18 articles including some sort of sweet taste evaluation [16,18,19,20,21,22,23,24,25,26,27,28,30,31,32,33].

Data analysis indicates that physical exercise increases sweet taste intensity. Based on before- and after-intervention results, the intervention groups showed significant differences for taste intensity ratings compared with the control groups. In general, such differences were also observed when comparing people who are physically active on a daily basis to those who are not [16,30,32]. A time effect was also observed. More specifically, sweet taste intensity is greater during exercise when compared with the before and after conditions, and sweetness ratings for a given stimulus increase in relation to the exercise duration [30]. Sweetness taste sensitivity was higher following a half marathon, with the absolute taste detection threshold of sucrose decreasing from 11.9 ± 1.0 mM (*p* = 0.14) to 7.7 ± 0.8 mM (*p* < 0.001) [26]. This sensitivity increase is attributed to a reduced absolute detection of sucrose following the exercise session [26]. While preference was the most commonly studied parameter, the results are contradictory. Although it seems clear that the preference for sweet solution increases both during and following (acute) exercise [19,20,23,24], one study presented different trends. When exercising and given a drink with a low concentration of carbohydrates, participants had a decreased preference for the drink compared with the control group [32]. Physical activity (chronic) seems to present different results. In fact, only one study presented meaningful results regarding a decrease in sweet preference; in this study, weekly physical activity levels were measured [16]. The group that was considered active reported a decrease in high sugary/fatty food preferences compared with the non- or less-active groups. It appears that physical exercise yields an immediate taste preference and acceptance of sugary foods following its completion, while diminishing this taste preference in everyday settings. Energy balance and overall glycogen depletion may influence sweet taste preference, sensitivity, and intensity following exercise, considering the effects of these factors on food choices and overall nutrition [34,35]. Knowing that chronic exercise is associated with weight reduction, and that weight loss yields positive results regarding sweet taste sensitivity, these factors may play a non-negligible role regarding taste preference [9,12]. The differences observed between exercise and physical activity regarding sweet taste preference may lie in these fundamentally different metabolic states, one being acute and one being chronic.

### 4.3. Salty

In this systematic review, 10 of the 18 selected studies included a testing of salty taste regarding intensity, sensitivity, or preference [17,19,21,22,24,27,29,30,32,33].

Overall, saltiness intensity ratings were lower during exercise compared with the before-exercise condition [30]. Saltiness intensity also seems to depend on the exercise duration, as the ratings for saltiness intensity decrease with exercise duration [30]. Significant results concerning salty taste sensitivity were present in only one study. Salty taste threshold sensitivity and physical activity levels showed a light correlation, trending towards an augmented salty taste sensitivity threshold [29]. The authors hypothesized that this effect comes from the sweat loss associated with physical activity [29]. As observed previously for the sweet taste, taste preference is one of the most studied variables when analyzing the effect of exercise on gustative perceptions. One key difference for salty taste compared with sweet taste is the all-round increase in preference. In fact, this increase is present during and following one exercise session [21,22,27], as well as when comparing people with different weekly physical activity levels; participants who were more active had a higher preference and overall acceptability of salty taste compared with less-active or non-active groups [17]. It is known that sweat loss affects overall sodium quantity within the exercising system [36]. This fluctuation has immediate and secondary effects on sodium preference and consumption following exercise and is well documented with rats [37] and within the human literature [21,22,27].

### 4.4. Bitter/Sour/Umami

Studies that evaluated sourness, umami, and/or bitterness were less common. Five such studies have been included in this systematic review, all of which produced significant results [18,19,24,27,32].

Overall, taste intensity for sourness decreased following the exercise condition when compared with sedentary or pre-exercise conditions [18,27]. Perceived taste intensity for umami was evaluated in only one study, and the results showed that the intensities of a high and low concentration of the umami tastant were perceived as higher in the exercising group compared with the control or non-exercising group [32]. Taste intensity results for bitterness were not significant in any study in this systematic review. For taste sensitivity in general, people who exercised were significantly better at identifying the umami taste compared with the control group; however, this result was reported in only one study [32]. Similarly, taste sensitivity results for bitterness and sourness were not significant in any study in this systematic review. A comparison of pre- and post-exercise conditions showed that physical exercise increases the preference for sourness [19,27]; this increase is also related to exercise status and/or sodium level [27]. Overall preference ratings for umami, at both low and high concentrations, were significantly lower for the exercise condition compared with control or non-active conditions [32]. Taste preference results for bitterness were not significant in any study in this systematic review. More studies on umami, sourness, and particularly bitterness are needed in order to draw stronger hypotheses regarding why certain phenomena are observed.

### 4.5. How Can We Measure Taste with a Novel Scope and How Can We Produce More Meaningful Results?

Over the last few decades, many discrepancies have been observed regarding energy intake and expenditure within the population [38]. Knowing that appetite and hunger are both responsible for eating behaviors, the accessibility of food and the reward-driven system associated with its consumption have created an obesogenic environment that promotes overconsumption and alters satiety signals [39]. Physical activity has been frequently linked to nutrition, highlighting its impact on appetite control, food choices, and intake [40,41]. The physiological basis of these changes has been explored and partly explained by the effect of physical activity on numerous appetite-regulating hormones, such as peptide YY-36, ghrelin, and glucagon-like peptide-1 [42]. It is currently known that people who are physically active tend to have lower cortical representation/activity within the food reward-related brain when shown images of high-caloric-density food compared with non-exercising participants [43]. Knowing that taste perceptions also regulate food preference and overall consumption, this systematic review aimed to clarify the state of the literature regarding the effect of exercise/physical activity on taste perceptions [6,44].

Food consumption and preference are highly regulated by palatability and macronutrient content; however, the chemosensory aspect of food also plays a significant role in its intake [45]. As taste exposure seems to be highly correlated with satiation, an increase in intensity and sensitivity to taste could lead to quicker meal termination, possibly regulating the quantity of food consumed onwards [46]. Although this could be observed regarding sweet and umami taste, salty taste would not be affected by this phenomenon; salty taste sensitivity and intensity are lowered with chronic and acute exercise, as seen in Table 4. As discussed in this review, exercise seems to have a chemosensory impact on overall taste perceptions. By exercising frequently, people could more quickly attain taste satiation, potentially lowering the consumption of highly tasty and energetic food and lowering the overall food quantity. The impact of physical activity in this matter could potentiate energy restriction by its effect on taste perceptions. Changes in taste perceptions and preferences, especially for the sweet taste, may lead to a weaker desire to consume foods that are hyperpalatable and rich in sugar. Currently, it has been documented that highly palatable foods tend to increase energy consumption [47]. A sharp decrease in the consumption of highly palatable foods, which are usually energetically dense, could decrease overall energy intake.

In the future, the usage of different tools for taste evaluation and detection remains a key aspect to consider in order to strengthen, improve, and expand our comprehension and testing abilities regarding this subject. As discussed previously in this review, most articles have studied the effect of physical activity on different taste sensory perceptions, but none have evaluated its impact with unique and novel equipment, such as the gustometer. Changes that could potentially occur without the participant’s full awareness could deeply change taste perception and detection. With this vision, the usage of an objective tool for assessment, as a new testing variable, in addition to questionnaires, scales, etc., issued by the participants could broaden our understanding and testing ability of the role of physical activity on gustative perceptions. In recent years, the use of a gustometer, such as the GU002 from Burghart Messtechnik, paired with an electroencephalogram has enriched our ability to test and assess gustative perceptions and thus refine our approach and understanding of this matter [48,49].

### 4.6. Strengths and Limitations

Most studies in this systematic review were published before the 2010s, with six published before the 2000s. Although this may seem like a methodological problem, the protocols and taste interventions were unexpectedly similar. Similarities were observed among most studies, such as the use of varying concentrations for the tastant of choice and, within the intervention details, the exercise selection and the type of taste test description. Nearly all studies in this systematic review, in which exercise was included as the intervention, used aerobic exercise as their preferred exercise method, with an intensity based on VO_2_max or a calculated/predicted maximal heart rate value [19,20,23,24,25,30,31,33]. Different scales were used to assess each parameter, but visual analog scales were the most common [16,20,22,24,25,27,28,30,32,33]. Considering that visual analog scales are considered by some as the gold standard for clinical experiments, offering more precise subjective results, their usage is usually associated with evidence of the effect of treatment [50,51]. The main discrepancies observed within this systematic review lie in the time of day for testing (in the morning, at noon, or in the evening) and the great variance between the pre-testing conditions and nutrition. Taste perceptions constantly change throughout the day, and the nutrition prior to testing may impact test results, as participants would have had different levels of hunger, appetite, and substrate utilization [52]. While most studies included in this systematic review had a normal-weight/healthy population [17,18,19,20,21,22,23,25,26,28,30,32,33], some had athletes [16,24,27], obese subjects [31], or partly clinically ill patients [29] as their testing subjects. Overall taste measures are known to be altered with weight gain and are usually negatively associated with higher BMI/adiposity [53,54]. Considering that these changes are crucial in understanding the role of adiposity/BMI in overall taste perceptions, future reviews that include a greater quantity of articles with BMI/adiposity as a central parameter should include detailed analyses regarding this matter.

## 5. Conclusions

To summarize, exercise and physical activity both exert significant effects on taste intensity, preference, and sensitivity, with the results and effects varying according to different modalities and the taste evaluated. Concerns regarding loss of smell and taste have emerged within the medical community. Thus, studies evaluating whether physical exercise can be a useful tool to enhance taste and smell could be critical for our understanding of this matter.

## Figures and Tables

**Table 3:** Risk of bias assessment.

Author, year	Risk of Bias				Applicability Concerns		
	Patient Selection	Index Test	Reference Standard	Flow and Timing	Patient Selection	Index Test	Reference Standard
Chrystal et al., 1995 [16]	(+)	(+)	(+)	(?)	(?)	(+)	(+)
Kanarek et al., 1995 [17]	(?)	(+)	(+)	(+)	(+)	(+)	(+)
Nakagawa et al., 1996 [18]	(+)	(+)	(+)	(?)	(+)	(+)	(+)
Horio and Kawamura, 1998 [19]	(?)	(+)	(+)	(+)	(+)	(+)	(+)
King et al., 1999 [20]	(+)	(+)	(+)	(+)	(+)	(+)	(+)
Leshem et al., 1999 [21]	(+)	(+)	(+)	(+)	(+)	(+)	(+)
Wald and Leshem, 2003 [22]	(+)	(+)	(+)	(+)	(+)	(+)	(+)
Horio, 2004 [23]	(?)	(+)	(+)	(+)	(?)	(+)	(+)
Passe et al., 2004 [24]	(?)	(+)	(+)	(?)	(+)	(+)	(+)
Havermans et al., 2009 [25]	(+)	(+)	(+)	(+)	(+)	(+)	(+)
Narukawa et al., 2009 [26]	(+)	(+)	(+)	(+)	(?)	(+)	(+)
Passe et al., 2009 [27]	(+)	(+)	(+)	(+)	(+)	(+)	(+)
Narukawa et al., 2010 [28]	(+)	(+)	(+)	(+)	(?)	(+)	(+)
Wen and Song, 2010 [29]	(+)	(+)	(+)	(+)	(?)	(+)	(+)
Ali et al., 2011 [30]	(+)	(+)	(+)	(+)	(+)	(+)	(+)
Martins et al., 2017 [31]	(+)	(+)	(+)	(+)	(+)	(+)	(+)
Feeney et al., 2019 [32]	(+)	(+)	(+)	(+)	(+)	(+)	(+)
Josaphat et al., 2020 [33]	(+)	(+)	(+)	(+)	(+)	(+)	(+)

(+)—Low Risk; (-)—High Risk; (?)—Unclear Risk.

**Table 4:** Impact of acute and chronic physical exercise on taste perceptions.

		Perceptions		
		Intensity	Sensitivity	Preference
**Taste**	Sweet	↑ (17, 18, 29)	↑ (25)	↑ (19, 20, 23, 24) ↓ (17, 29)
	Salty	↓ (28)	↓ (33)	↑ (18, 21, 22, 26)
	Sour	↓ (16, 26)	-	↑ (19, 26)
	Bitter	-	-	-
	Umami	↑ (29)	↑ (29)	↓ (29)

↓ = Decreasing results; ↑ = increasing results.
